# Pterostilbene Is a Potential Candidate for Control of Blackleg in Canola

**DOI:** 10.1371/journal.pone.0156186

**Published:** 2016-05-23

**Authors:** Joshua C. O. Koh, Denise M. Barbulescu, Phil A. Salisbury, Anthony T. Slater

**Affiliations:** 1 Department of Economic Development, Jobs, Transport and Resources, Grains Innovation Park, 110 Natimuk Road, Horsham, VIC 3401, Australia; 2 Faculty of Veterinary and Agricultural Sciences, University of Melbourne, Melbourne, VIC 3010, Australia; 3 Department of Economic Development, Jobs, Transport and Resources, AgriBio, Centre for AgriBioscience, 5 Ring Road, La Trobe University, Bundoora, VIC 3083, Australia; Universita degli Studi di Pisa, ITALY

## Abstract

Two stilbenes, resveratrol and pterostilbene, exhibit antifungal activity against *Leptosphaeria maculans*, the fungal pathogen responsible for blackleg (stem canker) in canola (*Brassica napus*). *In vitro* studies on the effect of these stilbenes on *L*. *maculans* mycelial growth and conidia germination showed that pterostilbene is a potent fungicide and sporicide, but resveratrol only exerted minor inhibition on *L*. *maculans*. Cell viability of hyphae cultures was markedly reduced by pterostilbene and SYTOX green staining showed that cell membrane integrity was compromised. We demonstrate that pterostilbene exerts fungicidal activity across 10 different *L*. *maculans* isolates and the compound confers protection to the blackleg-susceptible canola cv. Westar seedlings. The potential of pterostilbene as a control agent against blackleg in canola is discussed.

## Introduction

Canola (*Brassica napus*) is an oilseed crop ranked second in worldwide oilseed production (behind soybean), with an estimated total global production of 72.12 million metric tons in 2014–2015 [1]. *Brassica napus* is a member of the Brassicaceae family and originated through an interspecific hybridisation between *Brassica rapa* (AA genome, 2n = 20) and *Brassica oleracea* (CC genome, 2n = 18), resulting in an amphidiploid genome (AACC, 2n = 4x = 38) [2]. Canola is cultivated globally, and is found in cropping regions of North America, Europe, Asia and Australia [3, 4].

Blackleg, which is a phoma stem canker, is considered the most damaging disease to canola, being endemic to many canola-growing regions of the world [3, 5]. The most severe epidemics occur in Australia, where the disease almost wiped-out the fledgling canola industry in the early 1970s [6]. Blackleg is caused by the fungal pathogen *Leptosphaeria maculans*, a Dothideomycete which undergoes phases of biotrophic, necrotrophic and saprophytic growth during its life cycle [5, 7]. Sexual reproduction occurs on crop stubble that remains after harvest, via the production of ascospores which act as the primary inoculum. Ascospores are released from the pseudothecia during rainfall which generally coincides with the sowing period in late autumn in Australia. Ascospores germinate on cotyledons and leaves, and invade the plant via wounds or stomata. Following infection, the pathogen multiplies asexually causing phoma leaf spots, then a generally lengthy symptomless colonisation phase where the pathogen spreads from the leaf lesions through the petiole to the stem, where cankers can cause lodging and yield loss [5, 7].

The main approach to blackleg control is the breeding of resistance via the introduction of major and minor (quantitative) genes into canola cultivars. Major gene resistance, also known as qualitative or seedling resistance, is expressed at early stages of the plant development and remains active in adult plants [4, 8–10]. With a couple of exceptions [11, 12], qualitative resistance generally follows the “gene-for-gene” model in which interaction between the host major resistance (R) protein with a corresponding pathogen effector or avirulence (Avr) protein triggers the plant defence responses [8]. Although numerous R-genes have been mapped [7, 13, 14], only two have been cloned, *LepR3* and *Rlm2* [12, 15]. In contrast, seven avirulence genes (*AvrLm1*, *AvrLm2*, *AvrLm3*, *AvrLm4-7*, *AvrLmJ1/Lm5*, *AvrLm6* and *AvrLm11*) have been cloned in *L*. *maculans* [7, 13, 16, 17]. These effector genes are embedded within transposable element-rich regions in the genome allowing them to be easily lost or inactivated by repeat induced point (RIP) mutations during sexual reproduction [7, 18]. This genomic plasticity in combination with the strong selection for virulence imposed by resistant cultivars result in rapid evolution of virulent *L*. *maculans* populations. The rapid breakdown of resistance has been reported in commercial cultivars on two occasions. The first in France during 1996–1999 with resistance conferred by the *Rlm1* gene overcome within 5 years of release [19]. The second in Australia in 2003, with cultivars containing “sylvestris” resistance conferred by two major genes (*Rlm1*, *LepR3*) overcome within 3 years of release, resulting in up to A$10 million in losses [10]. It is accepted that without careful management, *L*. *maculans* will overcome major gene resistance and deployment of major genes needs to be integrated with other control measures.

Besides major gene resistance, quantitative or adult plant resistance which is attributed to many genes confers partial protection against *L*. *maculans* and is known to enhance the durability of major gene resistance in canola [9, 20]. However, breeding for quantitative resistance is more difficult. Although numerous studies have identified quantitative trait loci (QTL) for *L*. *maculans* resistance in *B*. *napus*, direct comparisons between QTLs are difficult due to differences in marker systems and disease pressures used in these studies [13]. In addition, the term “QTL” has been used ambiguously in the literature to describe traits explained by the major *R* locus [14].

Another effective approach to blackleg control is the use of fungicides. In Australia, fungicides currently in use against blackleg derive exclusively from group 3 (demethylation inhibitors, DMI) fungicides as field trials have shown that these are effective against blackleg [21, 22]. One of these fungicides is typically applied as a seed dressing (Jockey®, active ingredient fluquinconazole), one is applied in-furrow as a fertiliser amendment (Impact®, active ingredient flutriafol), and another is applied as a foliar fungicide (Prosaro® 420 SC, active ingredients prothioconazole and tebuconazole). However, reports of Jockey® tolerant *L*. *maculans* populations in recent canola field surveys [23] necessitates the continued development of novel fungicides against this fungus. This is crucial as *L*. *maculans* would likely continue to acquire tolerance to fungicides given its high evolutionary potential [7, 24].

Phytoalexins are low molecular weight, antimicrobial compounds produced in plants in response to pathogen attack. They have been proposed as a possible alternative to current fungicides [25–27]. One particular group of phytoalexins known as stilbenes have received tremendous interest due to its alleged health benefits for humans. Stilbenes occur across 33 plant families and comprise a relatively small group of phenolic compounds derived from the phenylpropanoid pathway via stilbene synthase (STS), which uses *p*-coumaroyl-CoA and cinnamoyl-CoA as precursors for the synthesis of the parent stilbenes, resveratrol and pinosylvin [28, 29]. Research has centred almost exclusively on resveratrol (3,5,4’-trihydroxy-*trans*-stilbene) which occurs in Vitaceae (grapevine) and Fabaceae (peanut), due to its antifungal, anticancer, antioxidant, anti-inflammatory and neuroprotective properties [30, 31]. In recent years, pterostilbene (3,5-dimethoxy-4’-hydroxy-*trans*-stilbene), a methoxylated analogue of resveratrol, has received increasing attention due to its superior pharmacokinetic properties [32, 33]. Both resveratrol and pterostilbene exhibit potent antifungal activities against a broad range of crop fungal pathogens such as *Botrytis cinerea*, *Fusarium oxysporum*, *Sclerotinia sclerotiorum*, *Plasmopara viticola* and *Septoria nodorum* [29, 34] but nothing is known about the effect of these stilbenes on *L*. *maculans*. The aim of this study was to investigate the antifungal properties of resveratrol and pterostilbene against *L*. *maculans*. We investigated if these stilbenes possess any fungicidal or sporicidal activity against *L*. *maculans* and whether they can protect canola seedlings from blackleg infection.

## Materials and Methods

### Fungal isolates and culture condition

The *Leptosphaeria maculans* isolates used in this study were cultured from individual ascospores released from infected *Brassica* stubble (S1 Table) and provided by Dr Angela Van de Wouw, University of Melbourne. Isolates were maintained on half-strength V8 agar (100 ml Campbell’s V8 juice, 1.5 g CaCO_3_ and 15 g agar per 1 L) in petri dishes in a 18°C temperature-controlled room under cool-white fluorescent light (Philips TLD 36W) and Grolux fluorescent light (Sylvania F36W/Gro) with a 20 h day/4 h night regime. A conidial suspension consisting of asexual spores (pcynidiospores) was prepared by flooding 13 day old sporulating cultures of the fungus with sterile distilled water, then filtering the conidial suspension through eight layers of cheese cloth. The concentration of the inoculum (spores/ml) was measured using a Brand® Neubauer improved haemocytometer (GmbH+Co.KG, Germany) and adjusted to concentrations specific to each experiment.

### Effect of stilbenes on *L*. *maculans* mycelial growth

Initial testing was done using *L*. *maculans* isolate D6, which is virulent towards the *Rlm3*, *Rlm4* and *Rlm9* major genes found in Australian cultivars [35]. Hyphal plugs (4 mm diameter) were excised from the edge of 13 day old cultures and transferred into the centre of 36 mm petri dishes containing 3 ml of half strength V8 agar amended either with resveratrol (Cayman Chemical, USA) or pterostilbene (Cayman Chemical, USA) from 50 mg/ml stock solutions in 100% EtOH to final concentrations of 25, 50, 100, 200 and 400 μg/ml. Control (solvent only) cultures had identical ethanol concentrations as treatments. Cultures were incubated at 18°C under the conditions described previously. There were three replicate culture plates of each treatment and the experiment was repeated once. Mycelial growth diameters (mm) were measured in two directions at right angle and recorded daily. After six days, mycelial growth was expressed as the percentage (%) of growth in the treatment relative to the control (100%) and the effective concentration (EC_50_) in which mycelial growth was reduced by 50% was calculated using GraphPad Prism® software version 6.07.

### Effect of stilbenes on *L*. *maculans* conidia germination

Conidial suspensions (100 spores/ml) of isolate D6 were spread in 1 ml aliquots onto 90 mm petri dishes containing 2% Bacto® water agar (BD Difco™, USA) amended either with resveratrol or pterostilbene at final concentrations of 50, 100 and 200 μg/ml. Control cultures (solvent only) had identical ethanol concentrations as treatments. Cultures were incubated in the dark at 25°C in a Memmert incubator (GmbH+Co.KG, Germany) and colonies formed after 10 days were counted and conidia germination was expressed as percentage (%) of number of colonies in the treatment relative to the control (100%). The experiment was done using three replicate culture plates and repeated once.

### Effect of pterostilbene on *L*. *maculans* conidia viability

Conidial suspension (1 x 10^7^ spores/ml) of *L*. *maculans* isolate D6 was amended either with 50 μg/ml pterostilbene or 0.1% v/v ethanol and incubated at room temperature for one hour. Aliquots of 200 μl were spread onto 90 mm petri dishes containing 2% Bacto® water agar (BD Difco™, USA). The remaining conidial suspension was diluted to a final concentration of 100 spores/ml and 200 μl aliquots were spread onto petri dishes as described above. The dilution of the sample was sufficient to prevent any residual activity of pterostilbene. The experiment was conducted using three replicate culture plates and repeated once. Culture plates were incubated in the dark at 25°C for 10 days and then examined for presence of viable colonies. High resolution grayscale images of culture plates were captured on a Gel Doc™ XR+ imaging system (Biorad, USA).

### Effect of pterostilbene on membrane integrity and cell viability

The effect of pterostilbene on the plasma membrane of *L*. *maculans* was investigated using SYTOX green staining. SYTOX green fluorescence increases significantly upon binding to nucleic acids but the dye can only enter cells when the plasma membrane is compromised [36]. *Leptosphaeria maculans* isolate D6 hyphae were grown in 50 ml half-strength potato dextrose broth (12 g potato dextrose per L) in 250 ml conical flasks from a starting concentration of 1 x 10^5^ spores/ml for 96 h at 25°C in the dark on an orbital mixer incubator (Ratek, Australia) at 180 rpm. Pterostilbene (final concentration 50 μg/ml) or ethanol (final concentration 0.1% v/v) was then added to the hyphae and the cultures were incubated at 25°C in the dark with gentle agitation. Aliquots (1 ml) of hyphae were taken at intervals of 2, 4 and 6 h for analysis. The experiment was done using five replicate culture flasks.

Hyphae were washed with sterile distilled water and resuspended in 1 ml sterile distilled water containing SYTOX green stain (Life Technologies, USA) at a final concentration of 0.5 μM. After 10 min, fluorescence of hyphae in microtiter trays were measured using POLARstar Omega microplate reader (BMG Labtech, USA) with excitation and emission wavelengths of 488 and 535 nm respectively, and visualised using an Olympus BX50 fluorescence microscope with an U-MWIBA3 filter (excitation 460–495 nm, emission 510–550 nm).

The viability of *L*. *maculans* hyphae was determined using fluorescein diacetate (FDA) staining. FDA is a cell-permeant dye which is hydrolysed by intracellular esterases to yield a fluorescent product (fluorescein) that is retained within the cell if the membrane is intact [37]. Hyphae cultures were prepared and sampled as described above. Hyphae were washed and resuspended in 1 ml sterile distilled water containing FDA stain (Sigma-Aldrich, USA) at a final concentration of 1 μg/ml. After 10 min, fluorescence of hyphae in microtiter trays was measured as described above.

### Effect of pterostilbene on *L*. *maculans* isolates

Based on the results from the experiments conducted on *L*. *maculans* isolate D6, a rapid screen was established to determine the antifungal activity of pterostilbene against ten isolates including D6, selected based on their virulence towards different major *R* genes currently deployed in commercial cultivars [35]. The effect of pterostilbene on the mycelial growth of ten *L*. *maculans* isolates (D1, D2, D3, D4, D6, D7, D9, D10, D13 and D14) was determined using experimental conditions described previously with the concentration of pterostilbene fixed at 50 μg/ml.

A second experiment then looked at the effect of pterostilbene on the conidia germination of five isolates (D3, D4, D6, D9 and D10), based on their sensitivity towards pterostilbene. The experiment was conducted using the conditions described previously with the concentration of pterostilbene fixed at 50 μg/ml and the concentration of inoculum 1 x 10^7^ spores/ml. High resolution grayscale images of culture plates were captured as described previously.

### Plant inoculation bioassay

A blackleg susceptible canola cultivar Westar [38] was used as the host for *L*. *maculans*. Seeds of Westar were sown in Jiffypots® (Jiffy USA) and seedlings were maintained under glasshouse conditions with a 16 h photoperiod at 22°C day/18°C night. Separate inoculums (1 x 10^7^ spores/ml) were prepared from *L*. *maculans* isolates D3, D6 and D9. Each lobe of the cotyledons of 8-day old Westar seedlings was wounded with a pair of forceps and 10 μl of inoculum amended immediately prior to inoculation either with 50 μg/ml pterostilbene or 0.1% v/v ethanol was applied to the wound sites. The experiment was conducted on 12 replicate seedlings and repeated once. Lesion development was assessed at 14 days post-inoculation with lesion diameter of less than 3 mm considered a resistant reaction and lesion diameter of more than 3 mm considered a susceptible reaction.

### Statistical analysis

Data were analysed for statistical significance using the statistical analysis software SPSS version 21 (IBM). Where appropriate, independent two-tailed Student’s *t*-test was used to compare the control against treatments.

## Results

### Effect of stilbenes on mycelial growth and conidia germination

**Fig 1:**
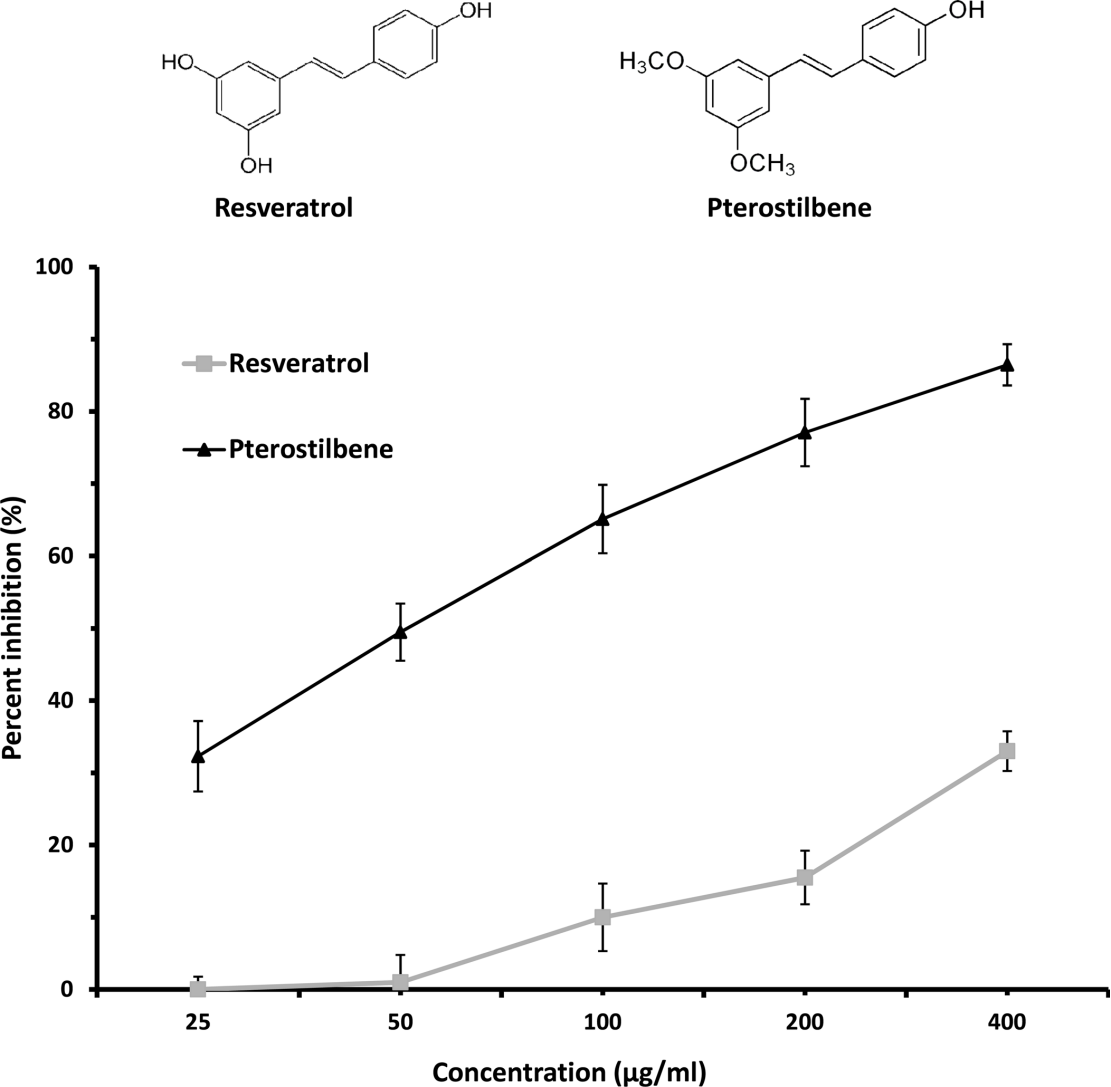
Inhibition of mycelial growth of *L*. *maculans* by the stilbenes resveratrol and pterostilbene. Hyphal plugs were cultured for six days on half strength V8 agar amended with resveratrol or pterostilbene at various concentrations. Mycelial growth is expressed as the percentage (%) of growth in the treatment relative to the control. Vertical bars represent standard deviation. Chemical structures of resveratrol and pterostilbene are shown above graph.

Pterostilbene inhibited mycelial growth markedly, with a concentration-dependent response observed where 32% of growth was inhibited at 25 μg/ml and up to 86% of growth was arrested at 400 μg/ml, when compared to the control (Fig 1). In particular, 50% of mycelial growth was inhibited at 50 μg/ml, which falls within the 95% confidence intervals (49.14–58.43 μg/ml) of the EC_50_ (53.59 μg/ml) calculated for pterostilbene. In comparison, resveratrol exhibited minor inhibitory effect on *L*. *maculans* mycelial growth, with 33% of growth inhibited at the highest concentration tested, 400 μg/ml (Fig 1).

**Fig 2:**
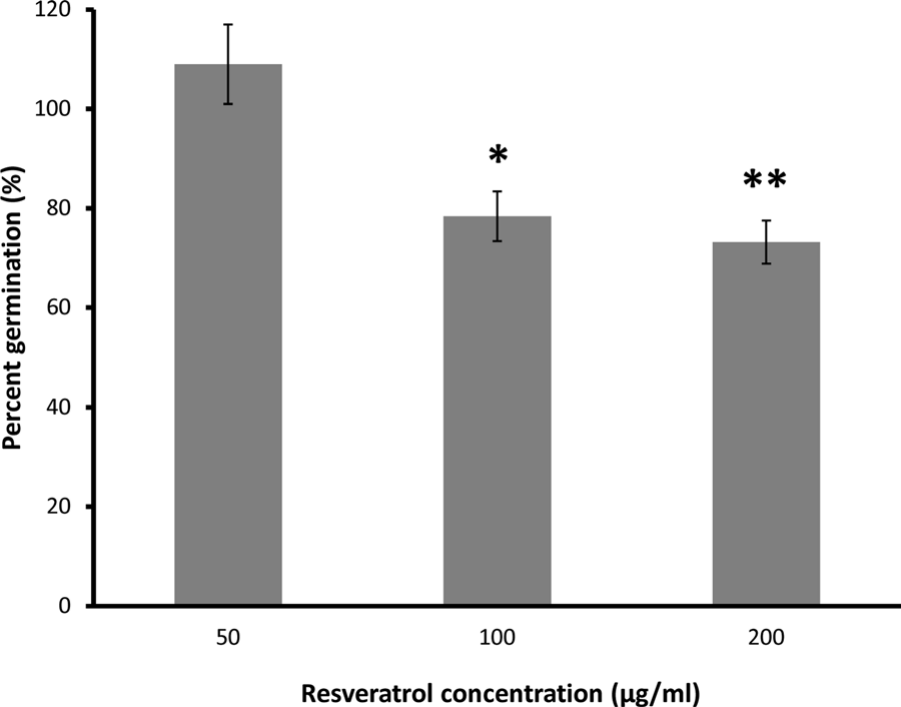
Conidia germination of *L*. *maculans* on 2% water agar amended with resveratrol at various concentrations. Colonies were counted after 10 days, and germination is expressed as percentage (%) of number of colonies in the treatment relative to the control. Statistical significance in Student’s *t*-test at *p*<0.05 (*) and *p*<0.01 (**) with degrees of freedom = 4. Vertical bars represent standard deviation.

In the conidia germination assay, resveratrol had no significant effect (*p*>0.05) on the germination of *L*. *maculans* conidia at 50 μg/ml when compared to the control (Fig 2). However, conidia germination was reduced by 22% at 100 μg/ml with a similar result of 27% germination reduction observed at 200 μg/ml. In contrast, for all concentrations tested (50, 100 and 200 μg/ml), pterostilbene completely inhibited conidia germination (data not shown). As such, pterostilbene was selected for further investigation as it showed potent antifungal activity against *L*. *maculans* in both mycelial growth and conidia germination studies whereas resveratrol had relatively minor inhibitory effects on *L*. *maculans*.

### Effect of pterostilbene on conidia viability

**Fig 3:**
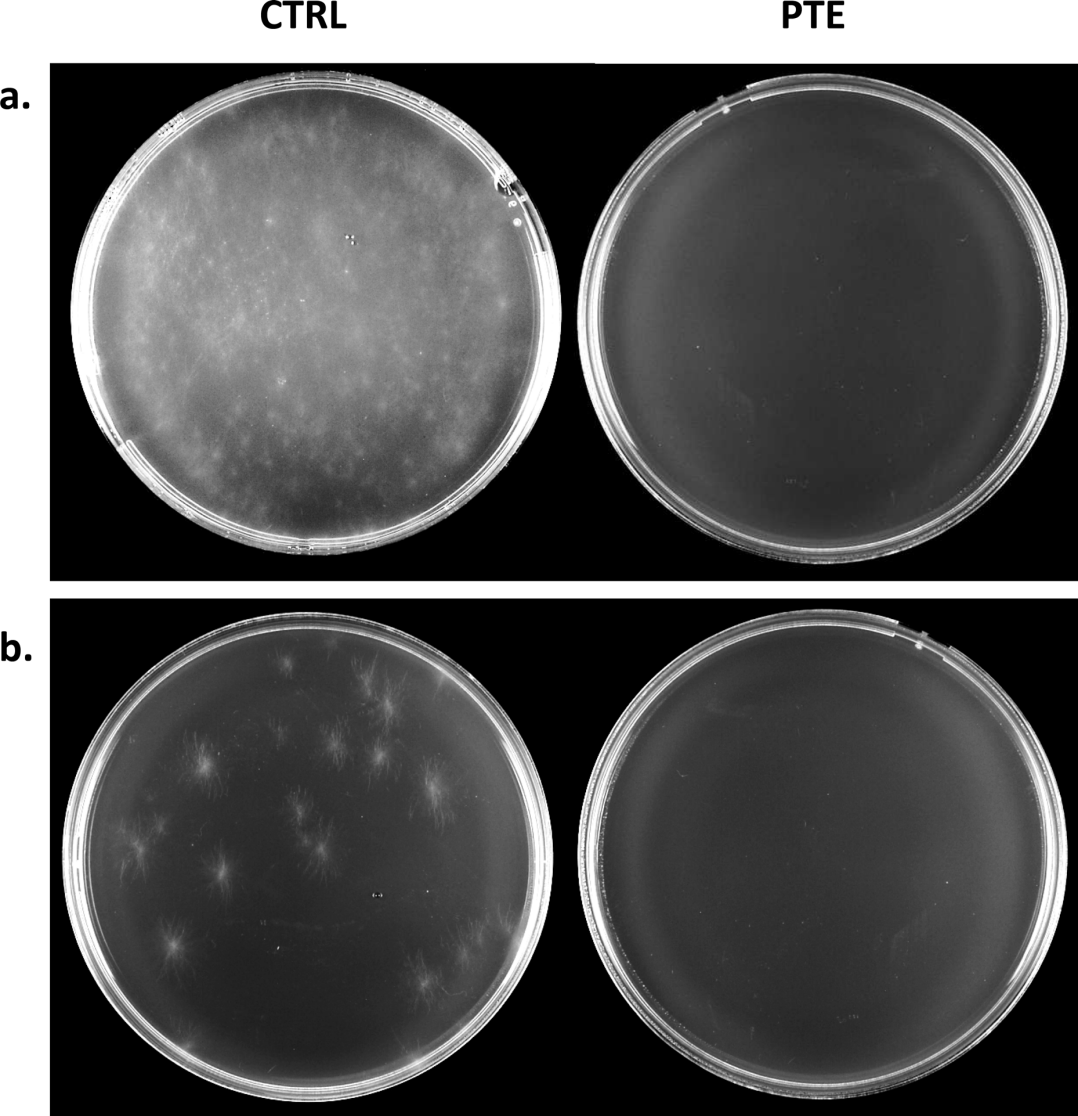
Sporicidal activity of pterostilbene against *L*. *maculans* spores. Growth after 10 days on 2% water agar of **(a)** 1 x 10^7^ spores/ml and **(b)** 1 x 10^2^ spores/ml cultures. Conidia (1 x 10^7^ spores/ml) were treated with 50 μg/ml of pterostilbene (PTE) or solvent (control, CTRL) and serially diluted to 1 x 10^2^ spores/ml.

At 50 μg/ml, pterostilbene was able to completely inhibit conidia germination at the high spore concentration of 1 x 10^7^ spores/ml (Fig 3). Importantly, no germination was observed even after pterostilbene was diluted to a negligible amount (less than 0.1 ng) in the conidial suspension, whereas germination and viable colonies were observed in the corresponding control (Fig 3), suggesting that pterostilbene killed the *L*. *maculans* spores.

### Effect of pterostilbene on membrane integrity and cell viability

**Fig 4:**
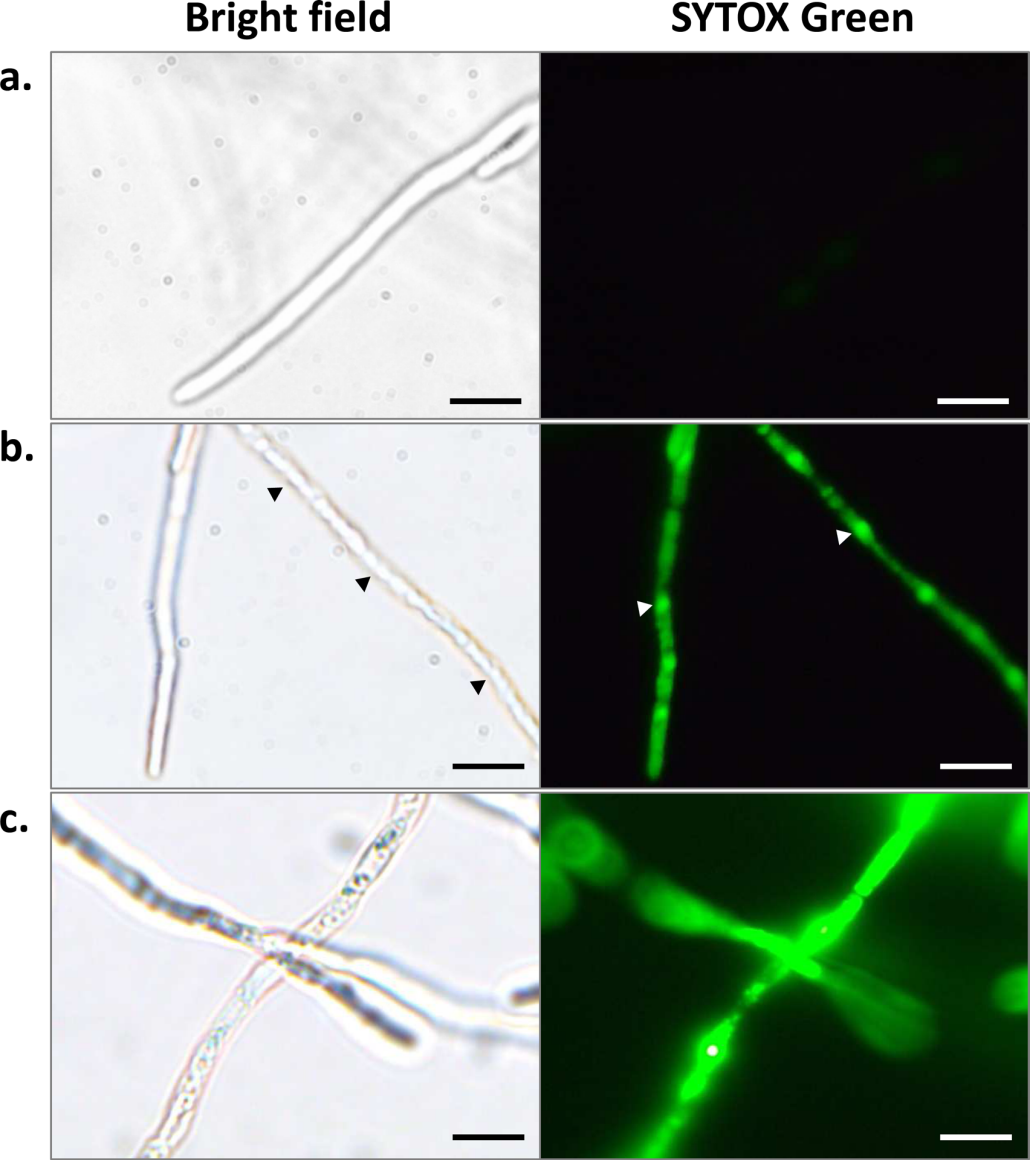
SYTOX green (0.5 μM) uptake into hyphae treated with 50 μg/ml pterostilbene. Bright field and fluorescent images for **(a)** control, **(b)** hyphae treated for 2 hours and **(c)** 4 hours. Black arrowheads indicate coagulation of the cytosol within the cytoplasm. White arrowheads indicate SYTOX green staining in the nuclei. Scale bar = 20 μm.

When *L*. *maculans* hyphae were treated with 50 μg/ml pterostilbene for 2 h, fluorescent nuclei were observed, indicating that membrane integrity was compromised (Fig 4). The nuclei of these permeabilized hyphae appeared intact but coagulation or aggregation of the cytosol could be seen in the cytoplasm. No fluorescence was detected in hyphae treated with just the solvent (control) (Fig 4). After 4 h incubation, a nonspecific and diffuse pattern of fluorescence was observed across the cell treated with pterostilbene (Fig 4). The nuclei were no longer intact and the cytoplasm of these permeabilized hyphae appeared granular.

**Fig 5:**
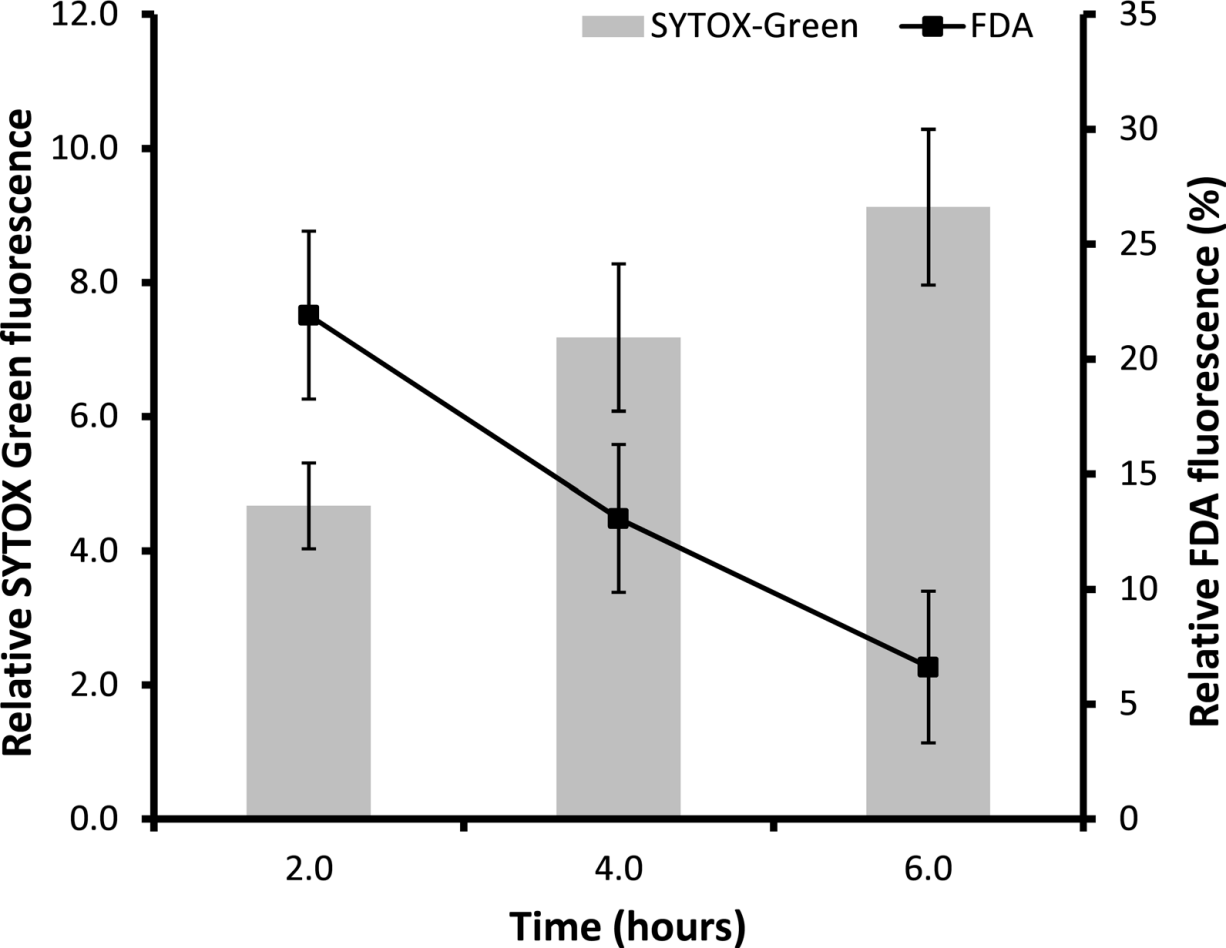
Permeabilization and cell viability of hyphae treated with 50 μg/ml pterostilbene. Uptake over time of 0.5 μM SYTOX green and 1μg/ml fluorescein diacetate (FDA) by hyphae cultures. Left axis: relative SYTOX green fluorescence (fold-change). Right axis: relative FDA fluorescence (%). Vertical bars represent standard deviation. All values were statistically significant at *p*<0.01 in Student’s *t*-test with degrees of freedom = 8.

The observation that membrane permeabilization increases over time was reflected in SYTOX green fluorescence measurement of hyphae treated with pterostilbene, with up to a 9.1 fold increase in fluorescence recorded after 6 h of incubation when compared to the control (Fig 5). The increases in membrane permeabilization corresponded with marked reductions in cell viability, as indicated by FDA staining (Fig 5). After 2 h of incubation, only 22% of cells were fluorescing indicating a reduction in viability of hyphae treated with pterostilbene and only 7% of cells were viable after 6 h of incubation, highlighting the fungicidal action of pterostilbene on *L*. *maculans* hyphae.

### Effect of pterostilbene on *L*. *maculans* isolates

**Fig 6:**
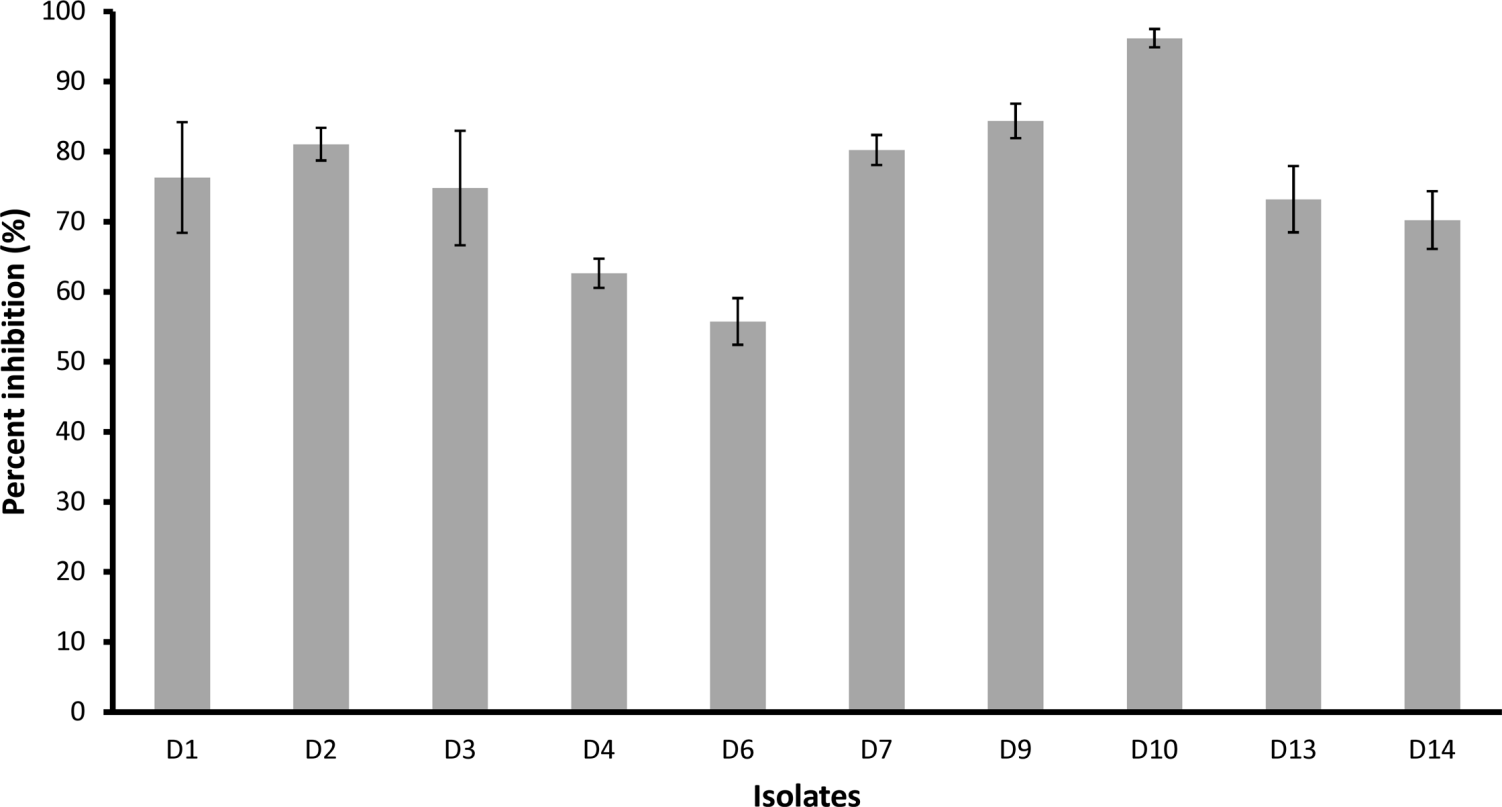
Inhibition of mycelial growth of ten *L*. *maculans* isolates by pterostilbene. Hyphal plugs were cultured for six days on half strength V8 agar amended with 50 μg/ml pterostilbene. Mycelial growth is expressed as the percentage (%) of growth in the treatment relative to the control. Vertical bars represent standard deviation. All values were statistically significant at *p*<0.01 in Student’s *t*-test with degrees of freedom = 4.

The effect of 50 μg/ml pterostilbene on mycelial growth of 10 isolates with different growth rates (S1 Fig) and avirulence genotypes (S1 Table) is shown in Fig 6. Pterostilbene was able to inhibit mycelial growth in all of the isolates tested, with 55%—96% growth inhibition observed across the isolates. Coincidentally, the first isolate tested, D6, was the least sensitive to pterostilbene (55% growth inhibition), with the majority of isolates recording a 70–80% inhibition in growth and isolate D10 was the most sensitive to pterostilbene with 96% growth inhibition (Fig 6). These results suggest that pterostilbene is extremely toxic to the *L*. *maculans* isolates, independent of their growth characteristics or ability to attack different major *R* genes.

**Fig 7:**
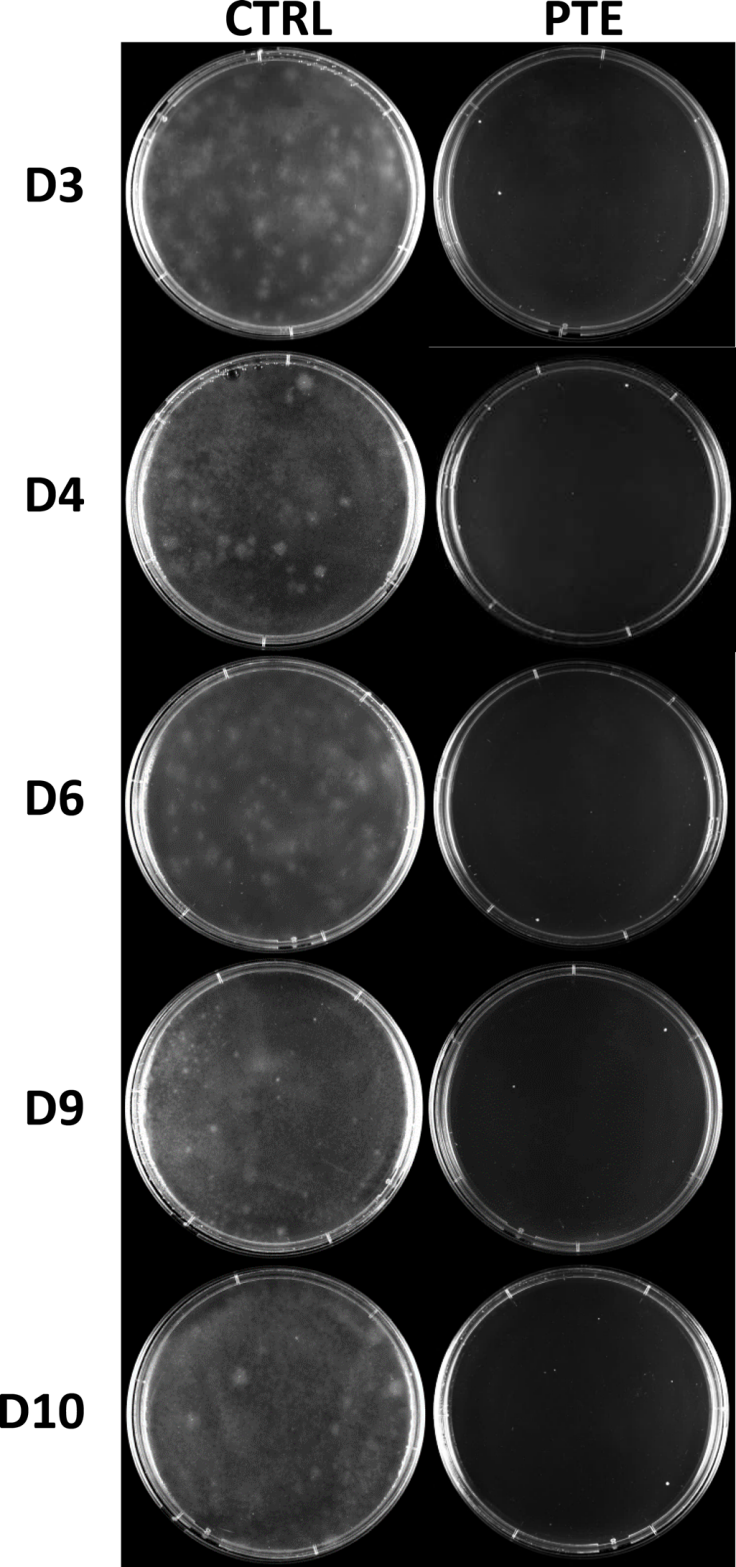
Inhibition of conidia germination of five *L*. *maculans* isolates by pterostilbene. Conidia (1 x 10^7^ spores/ml) germination after 10 days on 2% water agar amended with 50 μg/ml pterostilbene (PTE) or solvent (control, CTRL).

The effect of pterostilbene on the conidia germination of five isolates D3, D4, D6, D9 and D10, selected based on their sensitivity to pterostilbene (least sensitive to most sensitive: D6, D4, D3, D9, D10) is shown in Fig 7. Conidia germination from the high concentration of 1 x 10^7^ spores/ml was completely inhibited by pterostilbene across all five isolates, indicating that the fungicidal and sporicidal activities of pterostilbene against *L*. *maculans* are effective on a range of isolates.

### Plant inoculation bioassay

**Fig 8:**
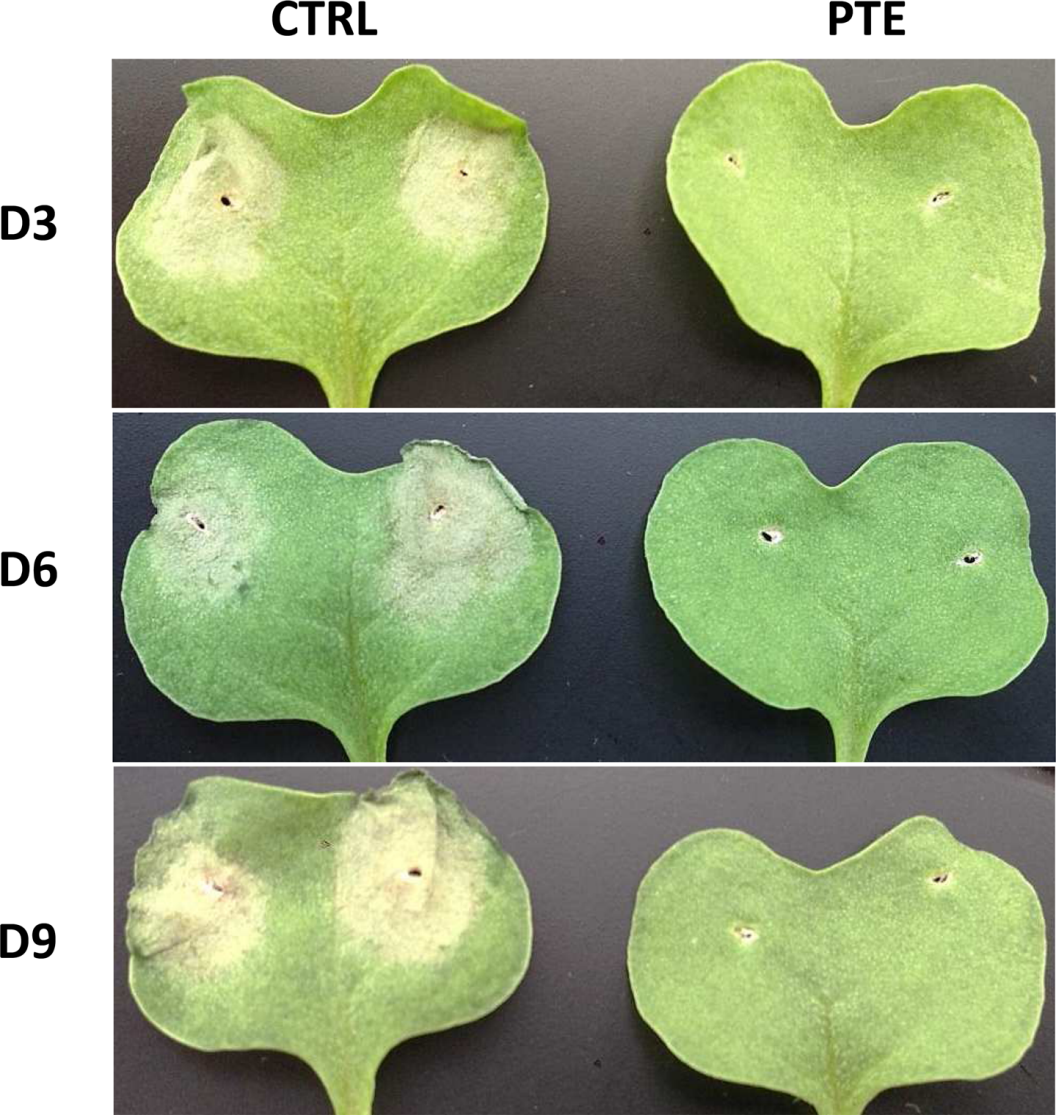
Plant inoculation bioassay on susceptible Westar seedlings. Cotyledons of Westar seedlings 14 days after wounding and inoculation with 10 μl of pcynidiospores (1 x 10^7^ spores/ml) from three *L*. *maculans* isolates amended with 50 μg/ml pterostilbene (PTE) or solvent (control, CTRL).

Pterostilbene was shown to protect a blackleg susceptible canola cv. Westar against *L*. *maculans* in seedling cotyledon inoculation assays. Fourteen days post-inoculation, the Westar seedlings treated with the control solution had completely succumbed to blackleg infection, with large lesions > 8 mm diameter visible on the cotyledons, but the Westar seedlings with pterostilbene appeared to be disease-free, with lesion development arrested at < 1 mm diameter or absent from the cotyledons (Fig 8). These results indicate that pterostilbene is not phytotoxic and conferred protection to Westar by inactivating *L*. *maculans*.

## Discussion

We report here the antifungal properties of resveratrol and pterostilbene against *L*. *maculans*. We show that pterostilbene, but not resveratrol, exhibits potent fungicidal and sporicidal activities against *L*. *maculans* and protects canola seedlings from blackleg infection.

*In vitro* studies examining the effect of resveratrol and pterostilbene on *L*. *maculans* mycelial growth and conidia germination showed that pterostilbene is a potent fungicide and sporicide but resveratrol only exerted minor inhibitory effects on *L*. *maculans*. This is not entirely unexpected, as studies in *Botrytis cinerea* showed that pterostilbene was fivefold more active than resveratrol in inhibiting *B*. *cinerea* conidia germination [39]. In particular, pterostilbene completely inhibited *B*. *cinerea* conidial germination at concentrations ranging from 52–60 μg/ml and trypan blue staining confirmed that fungal spores were dead [39]. Similar observations were made in our study where pterostilbene completely inhibited *L*. *maculans* conidia germination at 50 μg/ml and further testing confirmed its sporicidal activity. Pterostilbene also inhibited mycelial growth of *L*. *maculans* isolate D6 with an EC_50_ of 53.59 μg/ml (20.9 x 10^−5^ M) which is comparable to the activity of other phytoalexins at concentrations of 10^−4^ to 10^−5^ M [29]. In addition, pterostilbene showed antifungal activity against ten *L*. *maculans* isolates with different growth characteristics and avirulence genotypes, suggesting that the fungicidal action of pterostilbene could be wide-ranging against this fungus.

Differences in the fungicidal activity of resveratrol and pterostilbene have been attributed to the *in vivo* methylation of the hydroxystilbene group in pterostilbene [40, 41]. Studies have shown that the presence of methoxy groups improves the antifungal activity of stilbene derivatives, and methoxylation enhances membrane penetration by increasing the hydrophobicity of the compound enabling it to interact with lipophilic membranes. In contrast, the lower fungitoxicity of resveratrol may be due to it being more hydrophilic, which would limit transfer across lipophilic membranes [40, 41]. Methoxylated stilbenes also exhibit biological activities not seen in demethoxylated stilbene (e.g. resveratrol), for example, pterostilbene inhibits human recombinant cytochrome P450 CYP1A1 and CYP1B1, key enzymes involved in the detoxification of toxins or drugs [42], and also induces apoptosis in tumor cells via the caspase cascade in mitochondria [43]. Recent transcript profiling in *Saccharomyces cerevisiae* suggests pterostilbene exerts similar effects in yeast [44].

SYTOX Green staining of *L*. *maculans* hyphae treated with pterostilbene showed that cell membrane integrity was compromised. After 2 h, the cytosol appeared coagulated within the cytoplasm followed by granulation of the cytoplasm and cell death after 4–6 h. These observations are consistent with previous studies in *B*. *cinerea*, where pterostilbene and methylated stilbenes caused a marked alteration of *B*. *cinerea* conidial ultrastructures [40]. Specifically, pterostilbene caused a rapid degradation of the endoplasmic reticulum, nuclear and mitochondrial membranes, concurrent with a complete cessation of respiration. The cytoplasm accumulated into numerous vacuoles and the destruction of the conidium concludes with the disruption of the plasma membrane after 3 h [40].

The idea that phytoalexins could be deployed in crop protection is not new, but has met limited success because some of the tested fungi can overcome phytoalexins by detoxification [45, 46]. This has led to the development of a new generation of fungicides termed paldoxins (phytoalexin detoxification inhibitors), which are synthetic derivatives of phytoalexins that are resistant to fungal metabolism [46]. For example, paldoxins against *L*. *maculans* were derived from camalexin, a phytoalexin produced in *Arabidopsis thaliana* that could not be metabolised by *L*. *maculans* [47]. The manner in which pterostilbene acts on *L*. *maculans* is comparable to paldoxins in that *L*. *maculans* is likely unable to metabolise pterostilbene, a phytoalexin found mainly in grapevine (*Vitis vinifera*), *Vaccinium* berries (blueberry and deerberry) and wood from red sandalwood (*Pterocarpus santalinus*) [29, 48, 49]. In *V*. *vinifera*, susceptibility to *B*. *cinerea*, which is a major fungal disease, is linked to the fungus’ ability to metabolise stilbenes via enzymatic hydrolysis by laccases [50]. It is not known whether *L*. *maculans* possesses any laccase activity, but even if it does stilbenes are absent in the Brassicaceae. Therefore it is unlikely that *L*. *maculans* has the ability to breakdown these compounds and stilbenes could be an effective control.

Unlike paldoxins, however, which are designed to target specific fungal pathogens, pterostilbene displays broad-spectrum fungicidal activity against a large number of phytopathogens [29]. Pterostilbene exerts its antifungal activities via multiple modes of action [40, 44], thus making the risk of tolerance developing in *L*. *maculans* low. In addition, the sporicidal activity of pterostilbene against *L*. *maculans* make it an attractive candidate for development as a fungicide because the primary mode of blackleg infection in the field occurs through ascospores [3, 5]. Results in this study suggest that pterostilbene can protect canola seedlings against *L*. *maculans* by inactivating pcynidiospores (asexual spores), but further studies are required to see if the same activity is demonstrated against ascospores (sexual spores). Ascospores are more commonly isolated from infected canola stubbles in the field, but are difficult and time-consuming to obtain via *in vitro* crosses [51]. As chemical modifications can be done to increase the biological activity of pterostilbene [52, 53], studies examining the antifungal properties of pterostilbene derivatives against *L*. *maculans*, including their effect on ascospores, would be the next step in the development of a novel fungicide based on these compounds.

One of the main obstacles in the development of phytoalexins into fungicides is the high costs often associated with their production [27]. Fortunately, for the production of stilbenes, including pterostilbene, recent biotechnological advancements have made possible the biosynthesis of these compounds in metabolically engineered *Escherichia coli* [54–57]. In particular, a production titer of 2.3 g/litre resveratrol was achieved in *E*. *coli* with the use of a stilbene synthase (STS) gene from peanut (*Arachis hypogaea*) under carefully optimised conditions [57], making the production of stilbenes via *E*. *coli* fermentation commercially viable.

## Conclusion

Australian canola is in high demand globally for its use as a food-grade oil, for biofuel production and as a stock feed. Its production has increased significantly in recent years, with an annual production average of three million metric tonnes, accounting for 15–20% of the world’s export trade [58]. Against the backdrop of a thriving canola industry lies the constant threat of blackleg, with previous outbreaks costly and almost ended the burgeoning industry. The intense cultivation of resistant canola varieties without appropriate management has led to the rapid breakdown of resistance. Adding to the potential woes of blackleg control is the recent discovery of fungicide-tolerant *L*. *maculans* populations, thereby necessitating the development and deployment of novel fungicides. Results from our study present pterostilbene as a potential candidate for further development as a control agent for blackleg. Pterostilbene exhibits potent fungicidal and sporicidal activities against a range of *L*. *maculans* isolates. Further research on pterostilbene and other potential control agents in tandem with the discovery of new sources of resistance (qualitative and quantitative) would ensure that the canola industry continues to have the resources it needs to stay one step ahead of the blackleg menace.

## Supporting Information

S1 Fig Mycelial growth of *L*. *maculans* isolates on half V8 agar. Cultures were grown in 36 mm petri dishes and maintained at 18°C for six days under conditions described in the paper. Mycelial growth diameters (mm) were measured in two directions at right angle and averaged across three replicate culture plates. Vertical bars represent standard deviation.
(TIF)

S1 Table *Leptosphaeria maculans* isolates used in the study. (PDF)
