# Supplementary figures and images for: Pterostilbene Is a Potential Candidate for Control of Blackleg in Canola
Source: PLoS One. 2016 May 23;11(5):e0156186. doi: 10.1371/journal.pone.0156186 (PMC4877020; doi:10.1371/journal.pone.0156186)

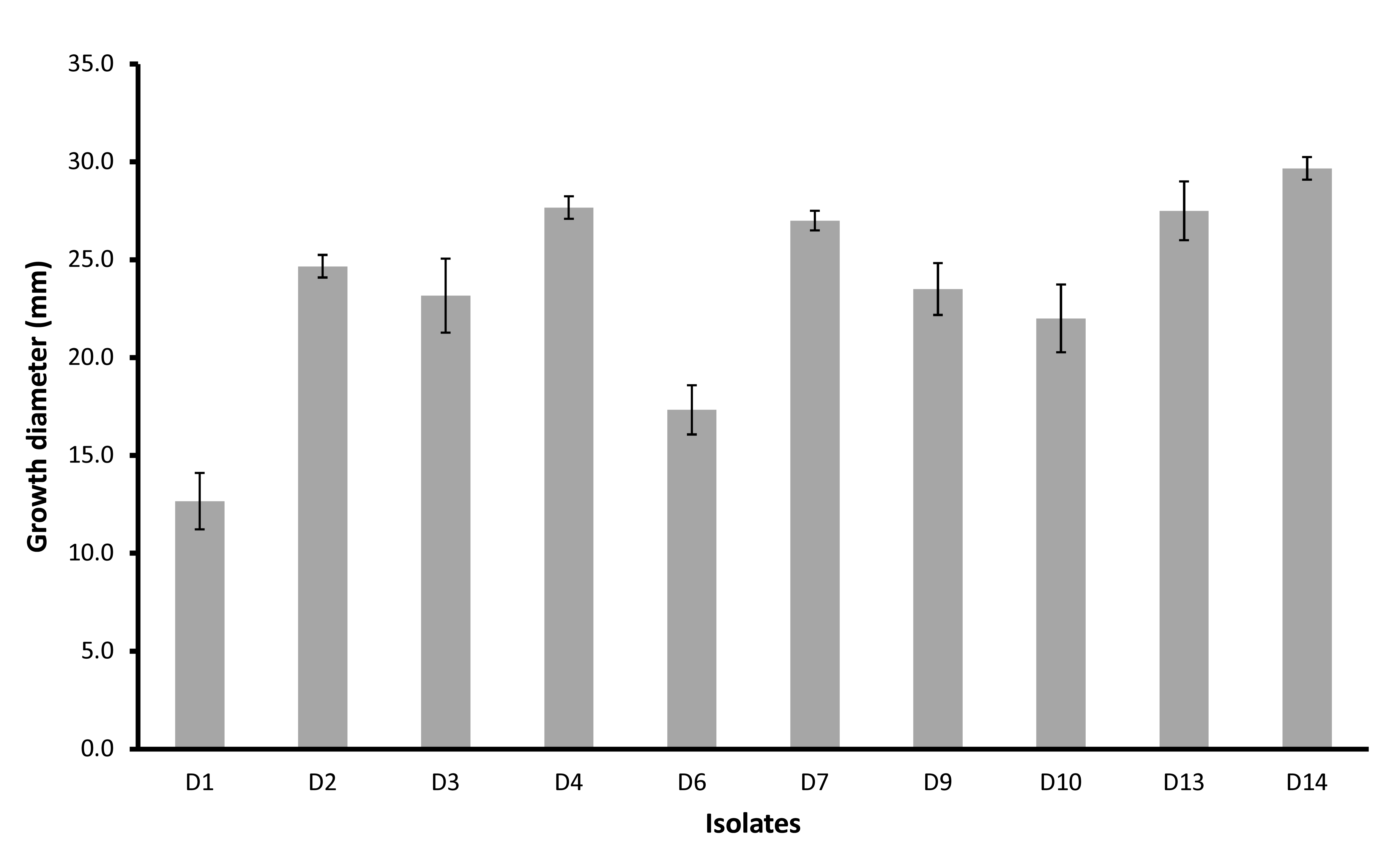

Supplement: S1 Fig — Cultures were grown in 36 mm petri dishes and maintained at 18°C for six days under conditions described in the paper. Mycelial growth diameters (mm) were measured in two directions at right angle and averaged across three replicate culture plates. Vertical bars represent standard deviation. (TIF) [file pone.0156186.s001.tif]
