# Supplementary material for: Pterostilbene Is a Potential Candidate for Control of Blackleg in Canola
Source: PLoS One. 2016 May 23;11(5):e0156186. doi: 10.1371/journal.pone.0156186 (PMC4877020; doi:10.1371/journal.pone.0156186)
Supplement: S1 Table — (PDF) [file pone.0156186.s002.pdf]

S1 Table. *Leptosphaeria maculans* isolates used in the study.

Isolate	Other names	Avirulence genes											*Avr* genotype
		AvrLm1	***AvrLm2***	***AvrLm3***	***AvrLm4***	***AvrLm5/J1***	***AvrLm6***	***AvrLm7***	***AvrLm8***	***AvrLm9***	***AvrLepR1***	***AvrLepR3***	
D1	IBCN13	-	+	-	-	+	+	-	-	+	+	+	*AvrLm2,5,6,9,LepR1,R3*
D2	IBCN15	-	-	-	-	+	+	-	+	-	+	+	*AvrLm5,6,8,LepR1,R3*
D3	IBCN16	-	-	-	-	+	-	-	-	-	+	-	*AvrLm5,LepR1*
D4	IBCN17	-	-	-	+	+	+	+	+	-	+	+	*AvrLm4,5,6,7,8,LepR1,R3*
D6	IBNC75	+	-	-	-	+	+	-	+	-	+	+	*AvrLm1,5,6,8,LepR1,R3*
D7	IBCN76, WA74	+	-	+	-	+	+	-	+	-	+	+	*AvrLm1,3,5,6,7,LepR1,R3*
D9	---	-	-	-	-	+	+	+	nd	-	+	-	*AvrLm5,6,7,LepR1*
D10	PHW1223	-	-	-	-	+	+	-	+	+	+	+	*AvrLm5,6,8,9,LepR1,R3*
D13	09SMW024	-	-	-	+	nd	+	+	nd	-	-	-	*AvrLm4,6,7*
D14	10SMJ023	+	-	-	-	+	-	+	nd	-	+	+	*AvrLm1,5,7,LepR1,R3*

Reference:

Marcroft SJ, Elliot, VL, Cozijnsen, AJ, Salisbury, PA, Howlett, BJ, Vande Wouw, AP. Identifying resistance genes to *Leptosphaeria maculans* in Australian *Brassica napus* cultivars based on reactions to isolates with known avirulence genotypes. Crop & Pasture Science 2012;63: 338-350.
